# The prognostic value of CXC subfamily ligands in stage I-III patients with colorectal cancer

**DOI:** 10.1371/journal.pone.0214611

**Published:** 2019-04-11

**Authors:** Xiangde Li, Qiulu Zhong, Danjing Luo, Qinghua Du, Wenqi Liu

**Affiliations:** Department of Radiation Oncology, The Second Affiliated Hospital of Guangxi Medical University, Nanning, Guangxi, China; University of South Alabama Mitchell Cancer Institute, UNITED STATES

## Abstract

**Objective:**

To investigate the value of CXC subfamily ligands in stage I-III patients with colorectal cancer, in order to find a new predictor for CRC patients.

**Methods:**

We used Gene Expression Omnibus (GEO) database to collect the gene expression of CXC subfamily ligands and corresponding clinical data. The survival analysis was performed by "survival" package of Rsoftware. The CRC patients’ DFS and the relationship between the expression levels of CXC subfamily ligands were evaluated by the univariate Cox regression analysis.

**Results:**

By using microarray data, there were 14 CXC subfamily ligands identified from dataset GSE39582. Seven CXC subfamily ligands were significantly correlated with DFS in CRC patients. (p<0.05),including CXCL1, CXCL3, CXCL9, CXCL10, CXCL11, CXCL13, and CXCL14. From multivariate Cox regression analyze, four CXC subfamily ligands (CXCL9, CXCL10, CXCL11, and CXCL13) were significantly associated with CRC patients’ DFS (all p<0.05). Three CXC subfamily ligands (CXCL10, CXCL11, and CXCL13) were significantly associated with CRC patients’ Overall survival (OS) (all p<0.05). Both CXCL11 and CXCL13 had the similar prediction values for DFS and OS.

**Conclusion:**

There were seven CXC subfamily ligands were significantly correlated with DFS in CRC patients. Different expression level of four CXC subfamily ligands (CXCL9, CXCL10, CXCL11, and CXCL13) and Three CXC subfamily ligands (CXCL10, CXCL11, and CXCL13) were related to CRC patients’ DFS and OS. There are still needs more experiments to confirm our conclusions. Next step we will make animal experiment about the genes in order to verified the predictive value of the CXC subfamily ligands.

## Introduction

Colorectal cancer (CRC) is one of the most highly mortality malignancies worldwide. Over half a million people died from colorectal cancer per year worldwide. Despite the progressed etiology of CRC and improved radiotherapeutic and chemotherapeutic regimens, there are still over half of colorectal carcinoma patients relapse or metastasis within 5 years. The pathologic stage of disease is crucial for the prognosis and treatment of colorectal carcinoma. But only depending on the pathologic stage of disease dose not enough to provide accurate prognosis for CRC patients. Only if patients’ outcome can predict accurately, can treatment be selected a most suitable regimens for individual patients, and avoided overtreatment. Thus, there are urgently to find new biomarkers which can improve the accuracy of prediction.

Chemokines are members of secretory small molecular cytokine proteins. They exerted their effect by specifically activating corresponding transmembrane G protein coupled receptors in tumor development and progression [1]. Some new studies pointed out metastasis were related to chemotaxis factors. Chemokines are divided into four subfamilies including CXC, CC, C, CX3C families. Up to now, there are more than 50 chemokines and 20 chemokine receptors have been identified. S. Gasperini pointed out CXC chemokines and their receptors not only could regulate angiogenesis, but also could stimulated tumor cells’ proliferation and metastasis. By connecting to chemokine receptor, chemokines could specifically moved to the stimulator, what calls chemolaclic movement. In the study of Zeelenberg[2], the expression of CXXR4 was essential for colorectal cancer with liver metastasis. And Ghadjar[3]also verified the relationship between the expression of CCR6 and colorectal cancer with liver metastasis. Kim[4]had verified over-expression of CXCR4 increased the risk of local recurrence in colorectal cancer, and indicated a poor prognosis. Chemokines might activated T cell to move directionally and then suppressed tumor’s growth [5]. Chemokines also could promoting the invasiveness of cancer cells by triggering integrin clustering and enhancing their adherence to extracellular matrix via their receptors [6,7]. Chemokines and their receptors had the potential to become the prognostic factors for CRC cancer. The mechanism of how the chemokines and their receptors adjust tumor development and progression asfollowed:1.Modulating angiogenesis; 2. Tumor specific immunity response activation; 3).Stimulating tumor-cell proliferation and metastasis [8]. Gunther[9] found that the expression of CCR7 predicted the lymph node metastasis. Meanwhile, Fukunaga[10] verified the expression of CXCR4 also leaded to lymph node metastasis. Chemokines became important biomarkers for evaluation of tumor in clinical, It also have potential to become predictors the local recurrence and metastasis in tumor.

Till now, there are few studies explan the chemokines how to lead the tumor relapse and metastasis in colorectal carcinoma. Chemokines have four subfamilies including CXC,CC,C,CX3Cfamilies[11]. Some studies pointed out chemokines had complex function on the oncobiology. One hand, chemokines could influence the life of tumor cell, and promoted tumor growth and metastasis, on the other hand, it suppressed tumor growth and metastasis by chemotaxis immunocompetent cell and inhibiting angiogenesis. Chemokines could inhibit angiogenesis, and CXCR3 was the main chemokin receptor in the process of inhibiting angiogenesis[12].In Mulle’s study, he pointed out CXCR4 only be found in cancer cells, they didn’t found in normal breast cells[13].Although there were some studies about the function of chemokines and their receptors for tumor metastasis, the sample size too small. We should pay closely attention to chemokines, try to illuminate chemokines how to make tumor cells metastasis, and find out a chemokine gene therapy and gene vaccine.It’s a potential way to cure cancer.

## Materials and methods

### Datasets preparation

Gene expression of CXC subfamily ligands and corresponding clinical data were obtained from the Gene Expression Omnibus (GEO) database (http://www.ncbi.nlm.nih.gov/geo/). All log2-transformed expression data of CXC subfamily ligands were obtained from Affymetrix human genome U133 plus 2.0 array (GSE39582, PMID: 23700391, DOI:10.1371/journal.pmed.1001453). The expression values of genes with multiple probes were calculated by using the median values of multiple probes. After filtering out CRC patients without disease-free survival (DFS) and clinical data, there were a total of 491 I-III stage patients with CRC.

### Statistical analysis

The survival analysis was performed by the “survival” package of R software (version 3.4.3).In dataset GSE39582, the association between the expression level of CXC subfamily ligands and CRC patients’ DFS was evaluated using the univariate Cox regression analysis. Those CXC subfamily ligands were considered to be significant if their *p*.values were less than 0.05. Using the median value of expression as the cutoff point, CRC patients were classified as low-expression or high-expression. Then, the selected CXC subfamily ligands were fitted in a multivariate Cox regression analysis to assess whether this risk score was independent of these clinical characteristics (*stage*, *age*, *gender*, *and adjutant chemotherapy*). Survival differences between low-expression and high-expression were assessed by the Kaplan-Meier estimator and log-rank test. The log-rank test, Cox regression analysis, and ROC analysis were considered to be significant if their *p*.values were less than 0.05.

### Functional enrichment analysis

To evaluate the functional implication of these CXC subfamily ligands (*CXCL9*, *CXCL10*, *CXCL11*, *and CXCL13*), functional enrichment analyses for GO and KEGG category were performed with the GeneCodis web tool (http://genecodis.cnb.csic.es/).[14–16]GO and KEGG category enrichments were based on the threshold of *p*.value<0.05. Significant enrichment results were visualized using R software (version 3.4.3).

## Results

### Identification of survival-related CXC subfamily ligands

By using microarray data, we identified 14 CXC subfamily ligands from dataset GSE39582. We further analyzed these 14 genes by the univariate Cox regression analysis in dataset GSE39582 (n = 491) (Table 1). Consequently, we identified seven CXC subfamily ligands that were significantly correlated with DFS in CRC patients (*p*<0.05, Table 1), including CXCL1, CXCL3, CXCL9, CXCL10, CXCL11, CXCL13, and CXCL14.

**Table 1:** Univariate Cox regression analysis of CXC subfamily ligands associated with disease-free survival in dataset GSE39582 (n = 491).

Gene Symbol	HR (95% CI)	Coefficient	*p*.value
**CXCL1**	0.846(0.749–0.957)	-0.167	0.008
**CXCL2**	0.814(0.620–1.068)	-0.206	0.137
**CXCL3**	0.850(0.755–0.957)	-0.163	0.007
**CXCL5**	0.932(0.848–1.026)	-0.07	0.150
**CXCL6**	0.906(0.801–1.025)	-0.099	0.116
**CXCL8**	0.926(0.839–1.022)	-0.077	0.127
**CXCL9**	0.828(0.744–0.923)	-0.188	0.001
**CXCL10**	0.819(0.731–0.917)	-0.200	0.001
**CXCL11**	0.872(0.797–0.955)	-0.137	0.003
**CXCL12**	1.015(0.863–1.194)	0.015	0.857
**CXCL13**	0.858(0.780–0.945)	-0.153	0.002
**CXCL14**	1.100(1.004–1.205)	0.095	0.040
**CXCL16**	0.921(0.730–1.161)	-0.083	0.485
**CXCL17**	0.916(0.694–1.210)	-0.087	0.538

### Multivariate Cox regression analysis for CXCL1, CXCL3, CXCL9, CXCL10, CXCL11, CXCL13, and CXCL14

Table 2 shows the multivariate Cox regression analysis results of seven CXC subfamily ligands in dataset GSE39582. Using the median value of these seven genes as the cutoff point, CRC patients were classified into high-expression and low-expression groups in dataset GSE39582 (low-risk/high-expression: 246/245). Four CXC subfamily ligands (CXCL9, CXCL10, CXCL11, and CXCL13) were significantly associated with CRC patients’ DFS (all *p*<0.05). Three CXC subfamily ligands (CXCL10, CXCL11, and CXCL13) were significantly associated with CRC patients’ overall survival (OS) (all *p*<0.05).

**Table 2:** Multivariate Cox regression analysis of seven CXC subfamily ligands, gender, age, stage, adjuvant chemotherapy, and survival in dataset GSE39582 (n = 492).

Gene Symbol	Variable	Disease-free Survival		Overall Survival	
		HR (95% CI)	*p-value*	HR (95% CI)	*p-value*
**CXCL1**	gene expression (high *vs*. low)	0.839 (0.598–1.175)	0.307	1.007 (0.722–1.404)	0.969
	age (≥65 years *vs*. <65 years)	1.010 (0.710–1.437)	0.956	1.851 (1.257–2.725)	0.002
	gender (female *vs*. male)	0.699 (0.495–0.987)	0.042	0.665 (0.474–0.932)	0.018
	tumor stage (III *vs*. II *vs*. I)	2.049 (1.413–2.972)	<0.001	1.655 (1.166–2.348)	0.005
	adjutant chemotherapy (no *vs*. yes)	0.884 (0.584–1.339)	0.561	1.517 (1.004–2.294)	0.048
**CXCL3**	gene expression (high *vs*. low)	0.870 (0.622–1.217)	0.417	1.048 (0.752–1.460)	0.784
	age (≥65 years *vs*. <65 years)	1.007 (0.707–1.433)	0.970	1.857 (1.260–2.735)	0.002
	gender (female *vs*. male)	0.703 (0.498–0.992)	0.045	0.666 (0.475–0.934)	0.019
	tumor stage (III *vs*. II *vs*. I)	2.062 (0.805–2.665)	<0.001	1.662 (1.173–2.355)	0.004
	adjutant chemotherapy (no *vs*. yes)	0.900 (0.595–1.361)	0.618	1.522 (1.010–2.295)	0.045
**CXCL9**	gene expression (high vs. low)	0.620 (0.441–0.872)	0.006	0.905 (0.651–1.258)	0.552
	age (≥65 years vs. <65 years)	1.014 (0.713–1.440)	0.940	1.845 (1.255–2.716)	0.002
	gender (female vs. male)	0.706 (0.500–0.995)	0.047	0.667 (0.476–0.934)	0.019
	tumor stage (III vs. II vs. I)	2.190 (1.503–3.191)	<0.001	1.676 (1.180–2.379)	0.004
	adjutant chemotherapy (no vs. yes)	0.964 (0.637–1.461)	0.864	1.535 (1.018–2.318)	0.041
**CXCL10**	gene expression (high vs. low)	0.548 (0.388–0.773)	0.001	0.696 (0.500–0.970)	0.033
	age (≥65 years vs. <65 years)	0.987 (0.694–1.402)	0.938	1.798 (1.222–2.646)	0.003
	gender (female vs. male)	0.712 (0.505–1.004)	0.052	0.675 (0.482–0.946)	0.022
	tumor stage (III vs. II vs. I)	2.206 (1.518–3.206)	<0.001	1.722 (1.212–2.444)	0.002
	adjutant chemotherapy (no vs. yes)	0.995 (0.657–1.505)	0.979	1.609 (1.067–2.429)	0.024
**CXCL11**	gene expression (high vs. low)	0.593 (0.421–0.835)	0.003	0.620 (0.443–0.867)	0.005
	age (≥65 years vs. <65 years)	0.999 (0.702–1.422)	0.997	1.797 (1.221–2.644)	0.003
	gender (female vs. male)	0.712 (0.610–1.223)	0.053	0.675 (0.481–0.945)	0.022
	tumor stage (III vs. II vs. I)	2.145 (0.958–2.140)	<0.001	1.728 (1.217–2.454)	0.002
	adjutant chemotherapy (no vs. yes)	0.975 (0.551–1.229)	0.907	1.636 (1.084–2.468)	0.019
**CXCL13**	gene expression (high vs. low)	0.593 (2.237–4.509)	0.003	0.620 (0.443–0.867)	0.005
	age (≥65 years vs. <65 years)	0.999 (0.720–1.452)	0.997	1.797 (1.221–2.644)	0.003
	gender (female vs. male)	0.712 (0.505–1.004)	0.053	0.675 (0.481–0.945)	0.022
	tumor stage (III vs. II vs. I)	2.145 (1.475–3.121)	<0.001	1.728 (1.217–2.454)	0.002
	adjutant chemotherapy (no vs. yes)	0.975 (0.643–1.480)	0.907	1.636 (1.084–2.468)	0.019
**CXCL14**	gene expression (high vs. low)	1.098 (0.787–1.533)	0.582	1.152 (0.828–1.605)	0.402
	age (≥65 years vs. <65 years)	1.015 (0.713–1.455)	0.935	1.847 (1.253–2.724)	0.002
	gender (female *vs*. male)	0.712 (0.505–1.005)	0.053	0.671 (0.478–0.939)	0.020
	tumor stage (III *vs*. II *vs*. I)	2.092 (1.444–3.032)	<0.001	1.663 (1.176–2.354)	0.004
	adjutant chemotherapy (no *vs*. yes)	0.900 (0.594–1.363)	0.619	1.489 (0.986–2.247)	0.058

### Survival comparisons for CXCL9, CXCL10, CXCL11, and CXCL13 between high-expression and low-expression in dataset GSE39582

**Fig 1:**
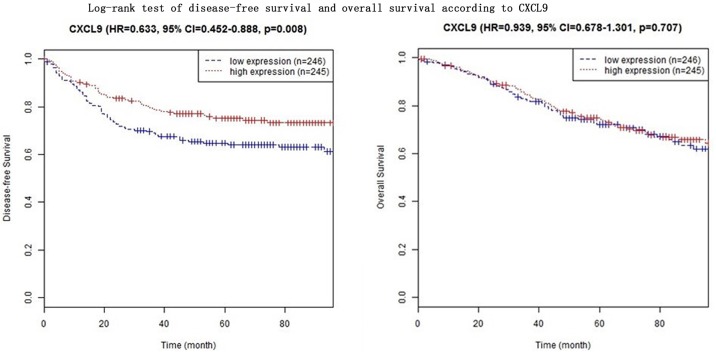
Log-rank test of disease-free survival and overall survival according to CXCL9.

**Fig 2:**
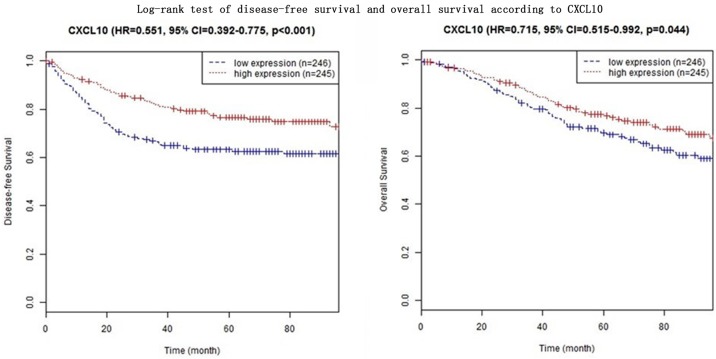
Log-rank test of disease-free survival and overall survival according to CXCL10.

**Fig 3:**
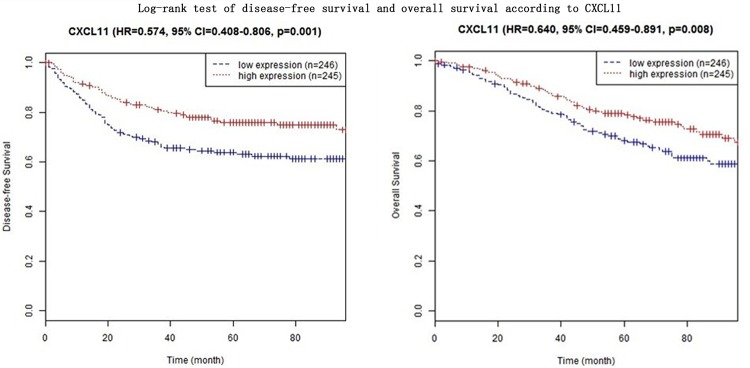
Log-rank test of disease-free survival and overall survival according to CXCL11.

**Fig 4:**
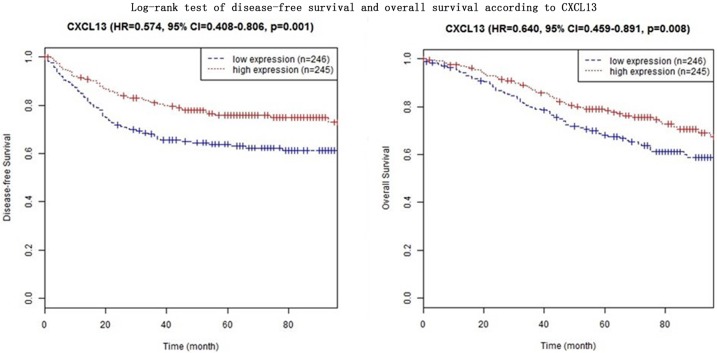
Log-rank test of disease-free survival and overall survival according to CXCL13.

**Fig 5:**
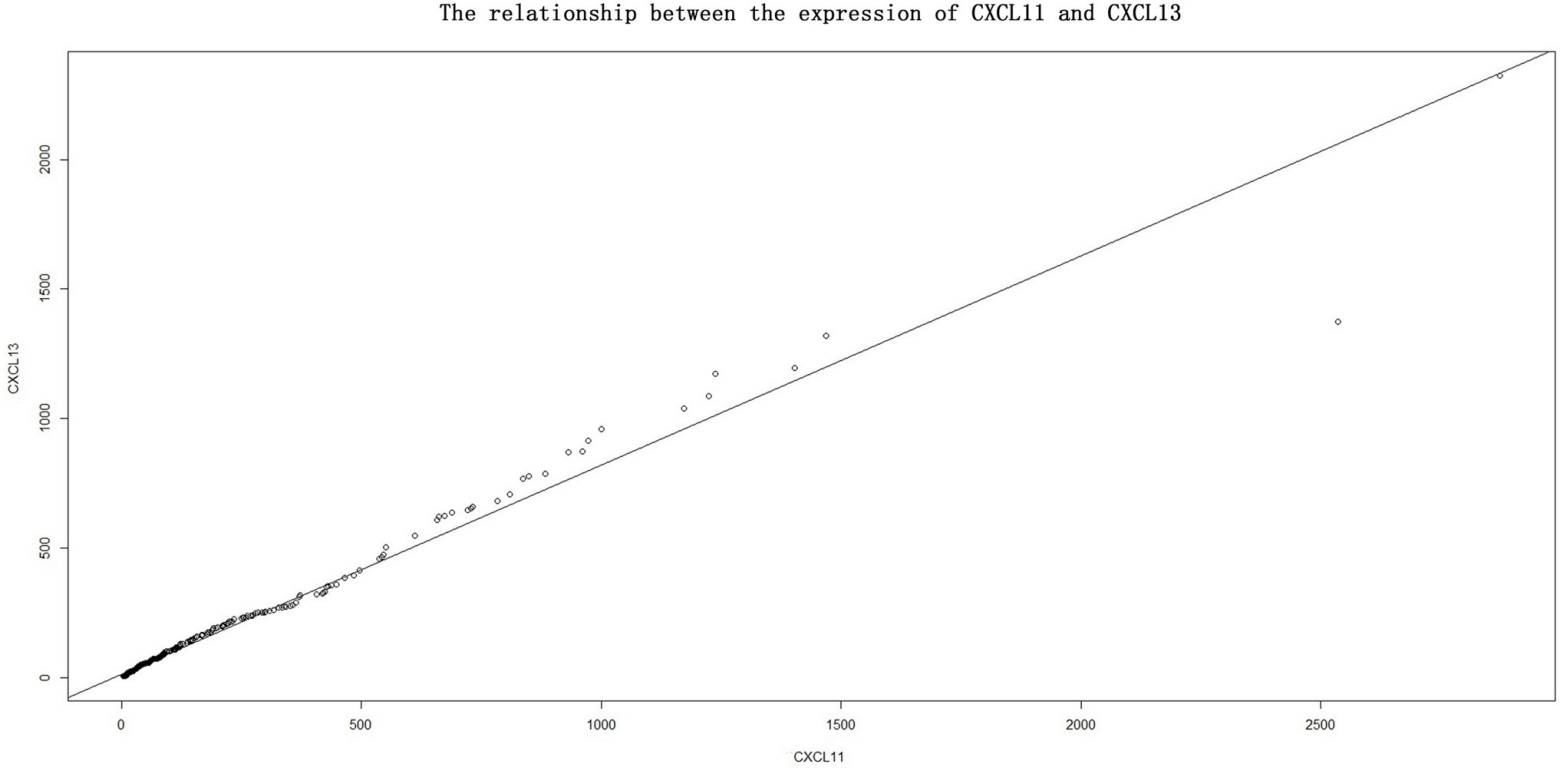
The relationship between the expression of CXCL11 and CXCL13.

In four CXC subfamily ligands (CXCL9, CXCL10, CXCL11, and CXCL13),the log-rank test showed that CRC patients with high expression significantly longer DFS than those with low expression (CXCL9: HR = 0.633, 95% CI = 0.452–0.888,*p* = 0.008; CXCL10: HR = 0.551, 95% CI = 0.392–0.775,*p*<0.001; CXCL11: HR = 0.574, 95% CI = 0.408–0.806,*p* = 0.001; CXCL13: HR = 0.574, 95% CI = 0.408–0.806,*p* = 0.001. Shown in Table 3 and Figs 1–4). In three CXC subfamily ligands (CXCL10, CXCL11, and CXCL13), the log-rank test showed that CRC patients with high expression significantly longer DFS than those with low expression (CXCL10: HR = 0.715, 95% CI = 0.515–0.992,*p* = 0.044; CXCL11: HR = 0.640, 95% CI = 0.459–0.891,*p* = 0.008; CXCL13: HR = 0.640, 95% CI = 0.459–0.891,*p* = 0.008. Shown in Table 3 and Figs 1–4). The expression of CXCL11 had a very high relevance with that of CXCL13 (r = 0.986, *p<*2.2e-16, Shown in Fig 5). And both CXCL11 and CXCL13 had the similar prediction values for DFS and OS (Shown in Table 3 and Figs 3 and 4).

**Table 3:** Log-rank test of disease-free survival and overall survival according to CXCL9, CXCL10, CXCL11, and CXCL13 expression in dataset GSE39582 (n = 491).

Risk group (n)	Disease-free Survival					Overall Survival				
	1-year	3-year	5-year	HR (95% CI)	*p*.value	1-year	3-year	5-year	HR (95% CI)	*p*.value
**CXCL9**										
**high expression (n = 245)**	90.3%	79.1%	75.1%	0.633 (0.452–0.888)	0.008	96.2%	84.5%	74.2%	0.939 (0.678–1.301)	0.707
**low expression (n = 246)**	86.2%	69.1%	64.9%			95.5%	82.2%	72.2%		
**CXCL10**										
**high expression (n = 245)**	92.4%	81.8%	76.6%	0.551 (0.392–0.775)	<0.001	96.3%	86.4%	76.7%	0.715 (0.515–0.992)	0.044
**low expression (n = 246)**	84.1%	66.5%	63.3%			95.4%	80.2%	69.6%		
**CXCL11**										
**high expression (n = 245)**	91.6%	81.1%	76.0%	0.574 (0.408–0.806)	0.001	97.1%	87.1%	78.3%	0.640 (0.459–0.891)	0.008
**low expression (n = 246)**	84.9%	67.1%	63.9%			94.6%	79.5%	68.1%		
**CXCL13**										
**high expression (n = 245)**	91.6%	81.1%	76.0%	0.574 (0.408–0.806)	0.001	97.1%	87.1%	78.3%	0.640 (0.459–0.891)	0.008
**low expression (n = 246)**	84.9%	67.1%	63.9%			94.6%	79.5%	68.1%		

Abbreviations: CI = confidence index, HR = hazard ratio, NA = Not Available.

### Functional enrichment analysis

**Fig 6:**
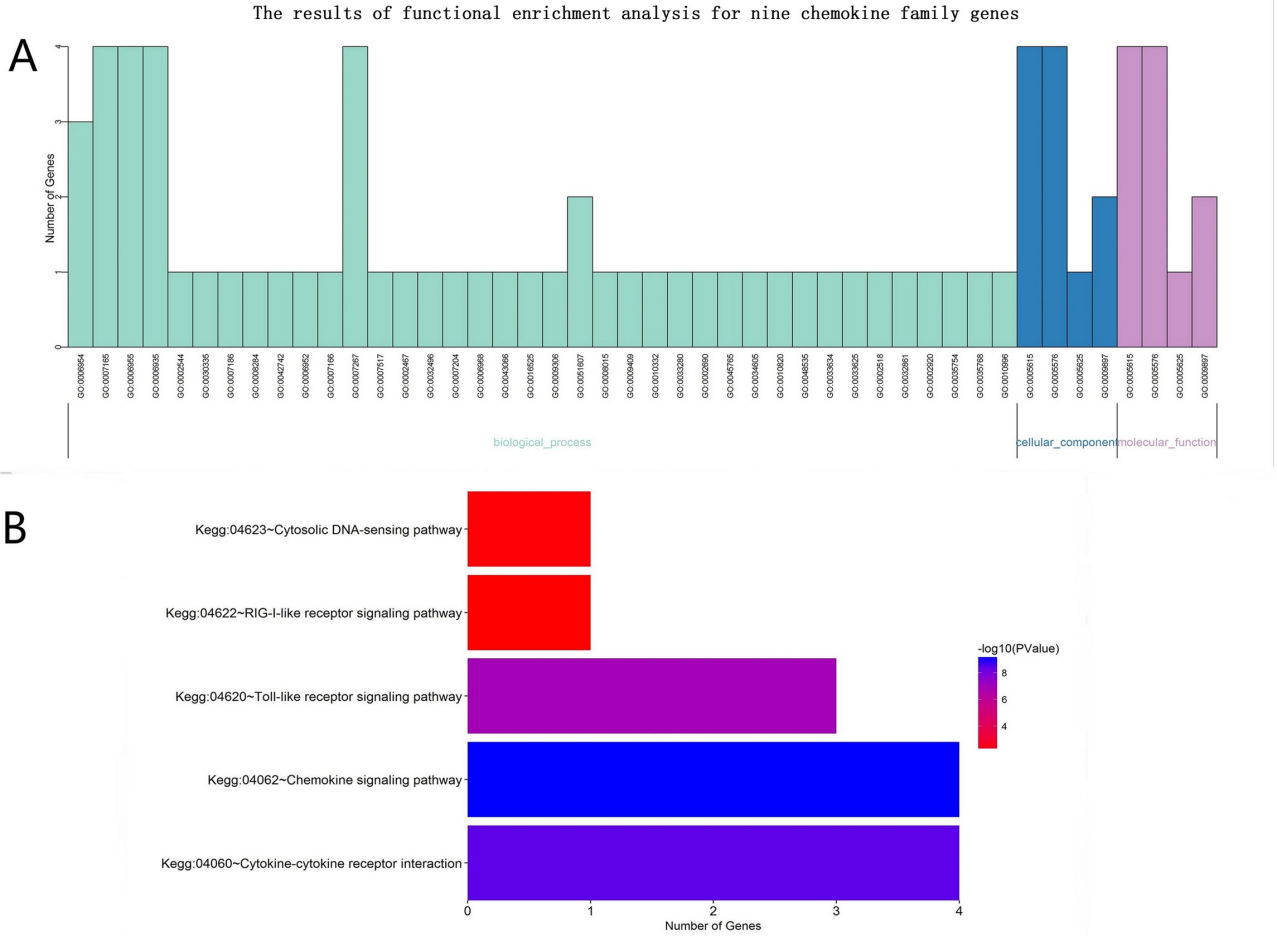
The results of functional enrichment analysis for nine chemokine family genes.

To explore the functional implication of four CXC subfamily ligands (CXCL9, CXCL10, CXCL11, and CXCL13), we performed functional category enrichment analysis to examine their functions. Functional enrichment analysis showed that nine chemokine family genes were significantly enriched in 46 GO terms and 4 KEGG pathways (Shown in Fig 6).

## Discussion

In our study, we found Seven CXC subfamily ligands were significantly correlated with DFS in CRC patients. And from the multivariate Cox regression analysis, there were four CXC subfamily ligands (CXCL9, CXCL10, CXCL11, and CXCL13) significantly associated with CRC patients’ DFS (all p<0.05). Three CXC subfamily ligands (CXCL10, CXCL11, and CXCL13) were significantly associated with CRC patients’ overall survival (OS) (all p<0.05).

In previous studies, more studies focused on CXCL12, CXCL14, while CXCL9 and CXCL10 had less research. As we know, CXCR3’s ligands included CXCL10 (IP-10) and CXCL11 (I-TAC), and few researches about CXCL9.CXCL10 was selected through stimulating leukemia U937cell lines from the DNA pool by Luster who comes from American [17]. It expressed by different cells, mediated the immune system to inhibit tumor angiogenesis, and then reduced tumor’s blood supply. In Hirano’s study, he found that CXCL-10 had high expression in HCC pathological, but through IHC he found that CXCL10 only expressed in HCC which had high Lymphocytes infiltrated [18]. CXCR4 was considered to be an important factor to regular angiogenesis. In Li W’s study [19], he showed that the expression of CXCR4 in HCC tissue more than other tissue such as paracancer tissue, cirrhotic liver tissue, normal liver tissue. And Schimanski showed that CXCL12 promoted CXCR4 receptor to translocation in Huh-7 HCC lines [20]. Not only CXCL12 combined with CXCR4, but also had high affinity with CXCR7. In Burns’ study, he pointed out CXCR4 also could combined with the CXCL12 of defective mice fetal liver cells, but the expression of CXCR4 and CXCL12 not all the same, that meant, excepted CXCR4, still had other new binding sites on the cell surface[21]. In our study, we found four CXC subfamily ligands (CXCL9, CXCL10, CXCL11, and CXCL13 were relevant to DFS. From Bandapall’s results [22], CXCL-chemokines were over-expressed in the tumor cells, especially CXCL1.And the level of CXCL1 was related to tumor growth,our results also justified this conclusion in homo species.But in our study, we further classified the seven CXC subfamily ligands which were significant statistical difference into high-expression and low-expression groups, and found CXCL9, CXCL10, CXCL11, and CXCL13) were significantly associated with CRC patients’ DFS (all *p*<0.05). CXCL10, CXCL11, and CXCL13 were significantly associated with CRC patients’ overall survival (OS). It means the level of CXC subfamily ligands’ expression influence CRC patients’ prognosis. CXCL8 also one of the most important CXC subfamily ligand. In Abhishek Kumar’s study[23], CXCL8 may promote migration through angiogenesis by upregulating VEGFA and it may the crucial role of CXCL8 during progression. To our study, there were no statistical significant of CXCL8 from dataset GSE39582, It may because in Abhishek Kumar’s study, they used LS174T human colon adenocarcinoma cells to establish an nude mice mode, different microenvironment in the nude mice influenced the results. Our study’s data were selected from Gene Expression Omnibus (GEO) database, and all data being selected were homo species, maybe colon adenocarcinoma cells in different microenvironment leads different results. Anyway, there are still many things need explored in chemokines.

CXCL9 activated T cells and NK cells through chemotaxis, and it cured cancer as anti-tumor agent. In previous studies showed high expression level of CXCL9 and CXCL10 in colorectal tumors could promoted lymphocytes infiltrate into tumors, and suppressed tumor cells’ growth [24,25]. In Ruehlmann’s study, he demonstrated that CXCL9 chemokine gene therapy which aided by small but non-curative doses of huKS1/4-IL-2 can suppressed the growth of murine colorectal carcinoma and inhibiting their pulmonary metastases [26].Zhenqian Wu et al showed the expression of CXCL9 was significantly associated with tumor differentiation, tumor invasion, lymph node metastasis, distant metastasis, and vascular invasion[27].K. Kawada and T.C. Walser similarly pointed out CXCL9 and/or CXCR3 antagonism meant better prognosis in melanoma and breast cancers, it also can inhibited the cancer metastasis[28,29].Zheng Jiang et al indicated that the expression of CXCL10 was an important prognosticator in stage II and III CRC, and it was relevant to CRC patients’ survival [30]. Sato E[31] had verified CXCL10 was an inhibitor to growth and metastases in tumor, and different expression level in CRC patients had different prognostic [32] In Jessicca D. Abron’s study, he investigated the role of CXCR3 in inflammation and colorectal cancer. The results demonstrated that polyphenols induce CXCR3 expression on regulatory T cells and increases CXCR3 ligands (CXCL9, CXCL10, CXCL11) in tumor microenvironment, it may become an important regulator in the treatment of CRC[33].

Our study used database to find the relationship between chemokines and CRC patients’ overall survival. From the Gene Expression Omnibus (GEO) database, we found that high CXC subfamily ligands expression have significantly longer DFS than those with low expression, CXCL11 and CXCL13 may become predictors for CRC patients. But our research based on the previous studies, the reliable of the results influenced by previous data, although our research used big database to find the relationship between chemokines and CRC patients’ OS, our study still have several limitations.

At last, from the research we verified that Seven CXC subfamily ligands were significantly correlated with DFS in CRC patients. Different expression level of four CXC subfamily ligands (CXCL9, CXCL10, CXCL11, and CXCL13) significantly associated with CRC patients’ DFS (all p<0.05). Three CXC subfamily ligands (CXCL10, CXCL11, and CXCL13) were significantly associated with CRC patients’ overall survival (OS) (all p<0.05). Our study still had limitation on account of the small sample and had selected bias in our study. There are still needs more experiments to confirm our conclusions. Next step we will make animal experiment about the genes in order to verified the predictive value of the CXC subfamily ligands.
